# Considering the Need to Expand the Indications for Wearable Defibrillator Therapy

**DOI:** 10.19102/icrm.2019.100707

**Published:** 2019-07-15

**Keywords:** External defibrillator, LifeVest, syncope, wearable defibrillator

## Dr. Milan comments

The case presented by Johnston et al.^1^ is remarkable for its successful negotiation of several clinical challenges. Of note, the prescription of a wearable cardioverter-defibrillator (WCD) in this patient with syncope is not routine; indeed, although the case is well-described, it is difficult at first glance to understand what clinical features led the clinicians to prescribe the WCD. Recognized historical high-risk features were largely absent in the history—these typically include the absence of a prodrome, injury associated with the syncope, family history of sudden death, and prior cardiac disease. It appears that the only high-risk feature in this patient was the exertional nature of one of her two episodes of syncope. According to established syncope risk scores such as Osservatorio Epidemiologico sulla Sincope nel Lazio (OESIL)^2^ and Short-term Prognosis of Syncope (StePS),^3^ she had only the abnormal electrocardiogram (ECG) (prolonged corrected QT interval at presentation) that signaled her higher risk. Yet, the clinicians managing this case prudently recommended a WCD—which may have saved the patient’s life.

The management of syncope can be difficult and the patient history is such an important component of the initial evaluation that it is difficult to challenge the decision-making of the treating physicians. I would argue that the physicians picked up on subtle historical features of the presentation that clued them in and made them uncomfortable with the thought of sending this patient home without some form of protection from ventricular arrhythmias. Given the myriad clues and nonverbal cues that we process when interviewing a patient and their family, it may be difficult to identify exactly what features convinced the treating physicians to prescribe the WCD. For this reason, until artificial intelligence systems develop “hairs on the backs of their necks,” physicians will likely continue to have job security.

The WCD^®^ 2000 system (Lifecor Inc., Blawnox, PA, USA) was approved in 2001 by the United States Food and Drug Administration through a premarket approval process and its name later changed to LifeVest^®^ in 2002, with eventual acquisition by Zoll Medical Corporation (Chelmsford, MA, USA) in 2006. The device is approved for “adult patients who are at risk for sudden cardiac arrest and [who] are not candidates for or [who] refuse an implantable defibrillator.”^4^ Technically, the present patient fits the bill. The WCD was approved based on demonstrated efficacy to successfully terminate ventricular arrhythmias without delivering a high rate of inappropriate therapies. However, large clinical trials to guide its use have not been forthcoming. The Vest Prevention of Early Sudden Death Trial and VEST Registry (VEST) study was negative for the reduction of sudden cardiac death (SCD) in the postmyocardial-infarction “waiting” period, but was likely underpowered and had a protracted enrollment period, during which time, the enrollment target was reduced. An unmeasurable challenge to the VEST study was also the availability of the WCD off-label, allowing physicians to readily prescribe a WCD for their postmyocardial-infarction patients with the highest risks and leaving only a lower-risk subset available for enrollment, thus making any benefit more difficult to detect. Despite the lack of hard clinical evidence to support the WCD’s use in syncope, this case demonstrates the importance of having the WCD in the cardiologist’s toolbox.

Considering for a moment the decision of which implantable cardioverter-defibrillator (ICD) might best benefit this patient, I would recommend a dual-chamber transvenous device. While I think that single-chamber ICDs are often preferred—especially in primary-prevention settings without pacing indications—in this patient who was already on escalating doses of antiarrhythmic medication, it might prove prudent to have the ability to pace the atrium in case there is spontaneous or drug-induced bradycardia. The subcutaneous ICD could be considered, but only after careful discussion of the positives and negatives regarding this patient who might need pacing in the future and who already experienced one successful antitachycardia pacing (ATP) episode in the first week after ICD implantation.

David J. Milan, md (dmilan@mgh.harvard.edu)^1^

^1^Cardiology Division, Massachusetts General Hospital, Boston, MA, USA

The author reports no conflicts of interest for the published content.

References1.JohnstonSLAshwathMLondonBTorgersonJTosicAOlshanskyBThe mysterious case of an athletic woman with recurrent syncope and a “normal” heart.J Innov Cardiac Rhythm Manage.20191073744374910.19102/icrm.2019.100701PMC7252779324777412.ColivicchiFAmmiratiFMelinaDDevelopment and prospective validation of a risk stratification system for patients with syncope in the emergency department: the OESIL risk score.Eur Heart J.2003249811819[CrossRef][PubMed]1272714810.1016/s0195-668x(02)00827-83.CostantinoGPeregoFDipaolaFShort- and long-term prognosis of syncope, risk factors, and role of hospital admission: results from the STePS (Short-Term Prognosis of Syncope) study.J Am Coll Cardiol.2008513276283[CrossRef][PubMed]1820673610.1016/j.jacc.2007.08.0594.United States Food and Drug Administration. Summary of safety and effectiveness data: P010030Available at: https://www.accessdata.fda.gov/cdrh_docs/pdf/P010030b.pdf. Accessed May 1, 2019

## Dr. Klein postulates

This 53-year-old woman was lucky to have been saved from drowning by her daughter and then to have fallen in the hands of experienced physicians who were not misdirected by debatable guideline adherence. Her complex premature ventricular complexes (PVCs) indicated a potential risk of life-threatening ventricular arrhythmia episodes, which could explain the repetitive loss of consciousness she experienced. Without a prior history of similar events and an inconspicuous family history of an inherited arrhythmia syndrome, more detailed risk stratification was mandatory in this case, particularly because of proven normal ventricular function, lack of heart failure, or significant ECG abnormalities. Prescribing the WCD here despite it not being mentioned in the syncope guidelines was, in my opinion, absolutely correct and was the only reasonable way to gain time to complete scheduled magnetic resonance imaging (MRI) diagnostics or further risk assessment. Early prescription of the WCD likely saved the life of this patient.

Using the WCD to protect this patient during risk stratification with the possibility of performing continuous ECG monitoring and analysis without being forced to hospitalize her is the most important benefit of the WCD in all cases and should always be the approach taken in cases with a potential risk of sudden arrhythmic death,^1,2^ regardless of the underlying disease. The WCD must not be considered a “therapy”; it allows for diagnostic procedures to be performed while ensuring protection of the patient in order to assist the physician with the decision to either confirm or defer the need for future ICD therapy. As such, it is hard to understand why the WCD is underrated and even neglected in some of the current guidelines.

An important component of the WCD is the response button that conscious patients can use to withhold shock delivery or to prevent inappropriate discharges for non–ventricular tachycardia (VT)/ventricular fibrillation (VF) signals. The VT/VF conversion rate is about 98%, which of course is not identical to survival of an arrhythmic event. Survival after successful defibrillation depends upon the resumption of regular cardiac function after a VT/VF event, regardless of whether defibrillation was performed by an ICD, WCD, or automated external defibrillator. Our longtime experience with WCD prescription has demonstrated that even very rapid VT events are often fairly well-tolerated in patients with normal ventricular function and allow for response button use for up to one hour, avoiding shock delivery with full consciousness.^3^ Even with new ICD programming algorithms, this would not be possible and may lead to unpleasant shock delivery. We also learned from the use of the WCD response button how often sustained VTs will terminate spontaneously.

The presentation in Johnston et al.’s **Figure 3** of the first WCD shock should not be described as simply a “failed” shock—that is, the first shock successfully terminated VT.^4^ However, after the first normal beat, sustained VT reinitiated and, after the second of potentially five shocks, the VT was permanently terminated.

When debating what could have been done differently, my answer is that the ICD was implanted too early and that WCD wearing should have instead been continued for at least three or even six months prior to potential ICD placement. The fact that another VT episode occurred shortly after ICD implantation does not mean that the patient continued to be at a long-term risk of sudden arrhythmic death. Actually, the authors mention that the patient remained arrhythmia-free with sotalol medication. Unfortunately, we do not learn how long the patient was followed after ICD implantation.

It was assumed that this patient suffered from myocarditis of most likely an acute nature, since arrhythmia symptoms occurred only two weeks before the drowning event in a seemingly so-far-healthy, symptom-free woman.

MRI scanning showed late gadolinium enhancement (LGE) deposits, which were interpreted as typical for nonischemic cardiomyopathy or fibrosis caused by viral myocarditis.^5^ One question to bring up here is: why was it considered enduring or nonperishable? With only one initial MRI scan, one could argue it would be impossible to confirm the stability or permanency of the LGE deposits. Not rarely, these deposits can disappear and the arrhythmic risk may become lower or even vanish.^6,7^ Therefore, we postulate that it would have been necessary to repeat MRI scanning after three or even more months under the protecting umbrella of the WCD. Only after further MRI scanning would the persistence of scar or fibrosis have confirmed the arrhythmia risk, providing enough information to implant an ICD. The Prolongation of Reverse Remodeling Period to Avoid Untimely ICD Implantation in Newly Diagnosed Heart Failure Using the WCD (PROLONG) study^8^ clearly demonstrated the usefulness of longer WCD prescription in order to justify or avoid ICD therapy.^8^ There are also numerous other studies that have revealed an improvement of left ventricular function and a disappearance of LGE deposits in patients with nonischemic cardiomyopathy or myocarditis.^9^

In summary, for this interesting case, our procedure would have been WCD prescription, MRI, and other diagnostic procedures (perhaps even myocardial biopsy), with prolonged WCD wearing lasting at least three months or even longer. Subsequently, only then would the decision for either single-chamber ICD or no ICD implantation be made.

Helmut U. Klein, md, fhrs, fesc (helmut.klein@heart.rochester.edu)^1^

^1^Clinical Cardiovascular Research Center, University of Rochester Medical Center, Rochester, NY, USA

The author reports no conflicts of interest for the published content.

References1.SalehiNNasiriMBiancoNRThe wearable cardioverter in non-ischemic cardiomyopathy: a US National Database Analysis.Can J Cardiol.201632101247.e11247.e6[CrossRef][PubMed]10.1016/j.cjca.2015.12.035269752242.WäßnigNGüntherMQuickSExperience with the wearable cardioverter-defibrillator in patients at high risk of sudden cardiac death.Circulation.20161349635643[CrossRef][PubMed]2745823610.1161/CIRCULATIONAHA.115.019124PMC49981243.KleinHUGoldenbergIMossAJRisk stratification for implantable cardioverter defibrillator therapy: the role of the wearable cardioverter-defibrillator.Eur Heart J.2013342922302244[CrossRef][PubMed]2372969110.1093/eurheartj/eht1674.JohnstonSLAshwathMLondonBTorgersonJTosicAOlshanskyBThe mysterious case of an athletic woman with recurrent syncope and a “normal” heart.J Innov Cardiac Rhythm Manage.20191073744374910.19102/icrm.2019.100701PMC7252779324777415.PiersSRDEveraertsKvan der GeestRJMyocardial scar predicts monomorphic ventricular tachycardia but not polymorphic ventricular tachycardia or ventricular fibrillation in non-ischemic dilated cardiomyopathy.Heart Rhythm.201512102106211410.1016/j.hrthm.2015.05.026260049426.HallidayBPBaksiAJGulatiAOutcome in dilated cardiomyopathy related to the extent, location, and pattern of late gadolinium enhancement.JACC Cardiovasc Imaging.2018 Sep 6pii: S1936-878X(18)30670-3[CrossRef][PubMed]10.1016/j.jcmg.2018.07.015PMC6682609302193977.SheppardRMatherPJAlexisJDImplantable cardiac defibrillators and sudden death in recent onset non-ischemic cardiomyopathy: results from IMAC 2.J Card Fail.2012189675681[CrossRef][PubMed]2293903510.1016/j.cardfail.2012.07.004PMC64816798.DunckerDKoenigTHohmannSBauersachsJVeltmannCAvoiding untimely implantable cardioverter/defibrillator implantation by intensified heart failure therapy optimization supported by the wearable cardioverter/defibrillator—the PROLONG study.J Am Heart Assoc.2017 Jan 1761pii: e004512[CrossRef][PubMed]10.1161/JAHA.116.004512PMC5523634280960989.ZecchinMMerloMPivettaAHow can optimization of medical treatment avoid unnecessary implantable cardioverter-defibrillator implantations in patients with idiopathic dilated cardiomyopathy presenting with “SCD-HeFT criteria”?Am J Cardiol.20121095729735[CrossRef][PubMed]2217699810.1016/j.amjcard.2011.10.033

## Dr. Gimbel explores

In this issue of *The Journal of Innovations in Cardiac Rhythm Management*, Johnston et al.^1^ present an interesting case of a middle-aged, healthy, fit, physically active woman with recurrent syncope who was managed within the context of an initially largely negative workup at a community hospital followed by discharge and subsequent referral to a tertiary care facility for further diagnostic testing and therapy. At the end of the manuscript, the authors pose several questions calling on the reader to consider some of the presenting features of the patient and deliberate on the validity of the patient’s subsequent management and therapy.

Regarding the first question, which inquires about the propriety of the WCD prescription, it seems quite reasonable to have implemented this technology to protect the patient during her outpatient transition to undergo further work-up and evaluation at a tertiary care facility. The authors noted that “current guidelines do not address the routine use of WCDs such as the LifeVest^®^ (Zoll Medical Corp., Chelmsford, MA, USA) in patients with syncope.”^1^ While this is true, the authors ask us to take a common-sense approach appealing to the Bayes theorem and the pretest probability that the patient’s recurrent symptomatic events were driven by a malignant arrhythmia despite a normal echo and no significant obstructive coronary disease at catheterization.

The patient’s initial presentation is informative and several high-risk features including syncope with exertion (while kayaking) and monomorphic triplets recorded during telemetry despite normalization of her potassium level are noted.^2^ The markedly prolonged corrected QT interval of 520 ms (Johnston et al.’s **Figure 1**) seen on the presenting ECG is also quite concerning, although not formally a “high-risk feature,” as repeated ECGs failed to show this finding.^2^ The use of nadolol 60 mg daily at discharge from the community hospital strongly suggested the clinicians believed long QT syndrome remained in the differential diagnosis. Interestingly, the patient did not report palpitations during her episodes of syncope, which can point toward cardiac syncope. Alboni et al. suggested in their publication, “when heart disease is absent, a cardiac cause is unlikely, unless palpitations precede syncope. In the latter case, the possibility that a tachyarrhythmia is the cause of syncope should be evaluated.”^3^ Despite the absence of reported palpitations, the clinicians in the present case appropriately maintained a high index of suspicion for arrhythmic syncope.

Perhaps the most disturbing high-risk element of the patient’s presentation is what is reported to have occurred during kayaking—namely, that the patient “fell out of the kayak and was pulled ashore by her daughter.”^1^ Certainly, some might characterize this as an episode of aborted sudden death, and this brings to mind a provocative editorial by Olshansky titled “Is Syncope the Same Thing as Sudden Death Except that You Wake Up?.”^4^ Had the daughter not been there to rescue the patient and pull her ashore, it is certainly not inconceivable that this patient may have drowned, leading to the final diagnostic maneuver being a gross, histologic, and perhaps molecular autopsy.^5,6^

One could argue the use of an external or internal loop monitor would be appropriate at discharge from the community facility (and perhaps readily paid for by an insurer), but, as Saklani et al. observed in their review of syncope, “an obvious disadvantage of a monitoring strategy is the potential for serious injury or death with a subsequent event, so it is generally inappropriate when [VT] or [VF] is suspected. Furthermore, the time course of recurrence is unpredictable.”^7^

Another point worth making is that the WCD offers the value of therapy while at the same time providing symptom–rhythm correlation by way of its diagnostic recording capability. While the value of the WCD is found principally in its ability to provide prompt life-saving tachyarrhythmic therapy, its value as a diagnostic tool to guide and refine subsequent therapy via the device’s monitoring facility has perhaps gone underappreciated.^8–10^

A referral to a tertiary care facility to provide a precise diagnosis is entirely appropriate, as ventricular tachyarrhythmias remained high in the differential as the cause of the patient’s syncope. In a patient with no apparent structural heart disease, some ventricular arrhythmias are not particularly life-threatening, while others can be lethal.^11^ Further, MRI is the next best step in the diagnostic work-up (perhaps in conjunction with genetic testing in this case); the 2014 European Heart Rhythm Association/Heart Rhythm Society/Asia-Pacific Heart Rhythm Society expert consensus statement on ventricular arrhythmias noted that “the presence of myocardial scar is more likely to be associated with poorly tolerated VT, hemodynamic collapse, degeneration to VF, and sudden death. In most cases, echocardiography can adequately demonstrate myocardial structure and function. If echocardiography is normal, more detailed imaging using cardiac MRI can exclude less clearly evident myocardial scar, arrhythmogenic [right ventricular] cardiomyopathy, nonischemic cardiomyopathy with preserved [ejection fraction], [hypertrophic cardiomyopathy], or cardiac sarcoidosis. It may also be helpful to reevaluate ventricular function when a patient with previously known [structural heart disease] presents with [sustained monomorphic VT].”^12^

In short, this patient presented with multiple high-risk features for cardiac syncope and, because of her required referral to a tertiary care facility for further diagnostic work-up (MRI) and therapy, I believe the use of the WCD as a bridge to protect her during transition to the tertiary care facility is entirely appropriate. In the case of long QT syndrome (which remained in the differential diagnosis), Owen et al. mentioned that “a WCD can provide an excellent, interim solution to protect a patient from the consequences of a life-threatening cardiac arrhythmia.”^13^

While “uncertain evidence and the uniqueness of a patient’s health care issues often make it difficult to identify the best course of care,”^14^ our first obligation as physicians is to think of the patient’s well-being. The fact that the off-label use of the WCD may not be covered by an insurance provider, with the costs subsequently borne by the patient is surely important, and the economic implications of prescribing a WCD should also be discussed with the patient.^15^ Ethically, however, the fact that the therapy might be expensive or even ultimately unaffordable for some does not absolve the physician from presenting the WCD as an option. Referral and prompt appearance at a tertiary care facility are likely to be expedited, keeping the wear time and thus cost of the WCD relatively low. In this case, I believe the use of the WCD to have been entirely appropriate.

Separately, considering the second question from the authors of why the patient did not pass out with a heart rate of 300 bpm, it is true that the rate of the wide complex tachycardia as recorded by the WCD is quite rapid, approaching 300 bpm. The answer to this question can only be speculative in nature, but, generally, “syncope during tachycardia is related to rate but modulated by the specific arrhythmia (supraventricular/ventricular), preload conditions, posture, left ventricular function, and adequacy of vascular adaptation.”^7^ The patient’s left ventricular function was normal as demonstrated by echocardiography and she had no obstructive coronary artery disease, both of which are findings that perhaps favor the maintenance of consciousness. In the recently published VEST trial, “a total of 69 participants in the device group aborted shocks by pressing the patient-response buttons during an alarm.”^16^ While the VEST manuscript does not make clear whether all such tachyarrhythmic events were VT, nor are readers told the rate of the rhythm, it seems plausible in this patient population, where the ejection fraction was about 28%, that many were able to tolerate sustained monomorphic tachycardia without syncope. In considering this second question from Johnston et al., one is reminded of Morady et al.’s report from more than three decades ago, which found “a sizable proportion of physicians are unaware that VT need not be associated with shock” and added that “more emphasis should be placed on making physicians aware that the differentiation of VT from paroxysmal supraventricular tachycardia [SVT] should be based on electrocardiographic findings and not on the patient’s blood pressure or clinical status.”^17^

Moving on to the third question, which asks us to consider between single-chamber, dual-chamber, and subcutaneous ICD therapy, perhaps surprisingly, one could ask whether this patient even needs an ICD. Might WCD use be able to be continued if the cardiomyopathy found on cardiac MRI is expected to resolve?^18^ This takes us to the question of just what was this patient’s diagnosis prior to ICD implantation. Nothing related in the Johnston et al. manuscript suggests the patient had a recent viral illness. However, this is not uncommon as, even in biopsy-proven myocarditis, the patient’s presenting symptoms can be quite variable, mimicking other cardiovascular conditions.^19,20^ While one might argue that the wide complex rhythm recorded by the WCD might be atrial flutter conducted aberrantly in a 1:1 fashion, this seems unlikely in light of the MRI findings.

Recalling the results of the MRI, which was obtained upon referral to the tertiary care facility, Johnston et al. mention that “the location of the patient’s scar suggested a substrate consistent with the morphology of her monomorphic [VT],” which involved the “basal-inferior and inferoseptal walls” (Johnston et al.’s **Figure 4**). Furthermore, “an MRI scan demonstrated the presence of delayed myocardial enhancement in the midmyocardium and epicardium, suggesting a nonischemic origin such as myocarditis, amyloidosis, sarcoidosis, or some other form of nonischemic cardiomyopathy. This patient’s scar was thought to be most consistent with a phenomenon of residual fibrosis from a past viral myocarditis.”^1^

In the case under consideration, the percentage of LGE extent is not specified for the reader, but the presence,^21,22^ extent,^23^ and LGE scar location^22,24^ can indeed be considered as important. Becker previously noted that “the amount of fibrotic burden, measured as the extent of LGE on [cardiac MRI], was described by various studies as a strong independent predictor for ventricular arrhythmic events.”^25^

One can recall from the echocardiogram that the present patient’s ejection fraction is normal. However, again, this is not an unusual finding in viral cardiomyopathies.^26^ Indeed, in their recent review and meta-analysis, Becker et al. noted that “these findings underscore that ejection fraction should be regarded as a readily available measure of substrate burden, particularly in patients with ischemic cardiomyopathy, but its usage should be reconsidered in patients with [dilated cardiomyopathy]. Cohort studies show that most patients with sudden death in this patient group have only a moderately reduced ejection fraction (> 35%) and, as recently demonstrated by Halliday et al., that the presence of mid-wall LGE identified a subgroup of patients at increased risk for SCD.”^25^ Turning to Halliday et al.’s work, these authors concluded that “a model based on the presence of septal LGE best predicted all-cause mortality. Whereas septal LGE was also associated with increased SCD events, the greatest risk was seen with concomitant septal and free-wall LGE. A model accounting for the greater risk associated with concomitant LGE in the septum and free-wall was most effective for SCD. Additionally, subepicardial or multiple patterns of LGE were associated with a high-risk of SCD events.”^27^ Though nothing is provided in the history of the current patient to suggest a recent viral illness, the patient’s MRI findings are consistent with a viral cardiomyopathy. A normal ejection fraction is frequently seen in patients with viral cardiomyopathy, and the presence and pattern of LGE^27,28^ and manifest VT clearly indicates a patient at risk for further VT and SCD. Clearly, some type of therapy for the patient’s ongoing risk of VT is indicated.

While emerging as the reference noninvasive diagnostic standard, cardiac MRI is still a work in progress^29^ and endomyocardial biopsy (EMB) remains the gold standard method for diagnosis.^20,26^ Consideration might have been given to endomyocardial biopsy (EMB) prior to ICD implantation if a viral myocarditis was suspected.^20^ The authors did highlight that the interpretation of the cardiac MRI image was “most consistent with a phenomenon of residual fibrosis from a past viral myocarditis.”^1,30^ EMB has not been widely embraced, and a dated consensus statement previously suggested that “EMB may be considered in the setting of unexplained ventricular arrhythmias only in exceptional cases in which the perceived likelihood of meaningful prognostic and therapeutic benefit outweighs the procedural risks.”^31^ Elsewhere, advocates of a combined cardiac MRI–EMB approach claimed that the “treatment of myocarditis patients essentially stands on symptomatic treatment of signs and symptoms of cardiac disease and of hemodynamic impairment [as well as] on etiology-directed treatment,”^32^ but further cautioned that “EMB should never be withheld when it has the potential to change the therapeutic strategy, since it allows [for the] detection of giant-cell or eosinophilic myocarditis and [the] exclusion of infectious agents or viral genome in the myocardium of patients who may be candidates for immunosuppressive treatments.”^20^

Nevertheless, “[the] indication for [ICD implantation] is controversial, because myocarditis may heal completely. Bridging using a LifeVest^®^ [(Zoll Medical Corp., Chelmsford, MA, USA)] in patients with myocarditis and severe ventricular arrhythmia [(ie, VT, VF)] could solve the transient problem.”^20^

Thus, rather than moving immediately toward implantation of an ICD, another option could have been to continue the WCD prescription until the chronicity of the myocarditis (and ongoing risk for VT/VF/SCD) was firmly established. Finally, given the presentation of the patient in question (ie, syncope/arrhythmic), a “negative” cardiac MRI (no LGE) would not preclude the clinician from proceeding with EMB, as cardiac MRI in an arrhythmic presentation may not be particularly sensitive for myocarditis.^19^ In the present case, while the patient’s cardiac MRI findings appear consistent with a prior myocarditis, an understanding of the onset and chronicity of the myocarditis may have been improved with the performance of EMB. Whether this would have led to a delay or even omission of ICD implantation is entirely speculative and unknown. Peretto previously remarked that, “even after inflammation recovery, scar-related ventricular arrhythmias can occur any time during follow-up”^24,26^ and, thus, EMB, irrespective of the findings, may not have materially impacted the choice of whether to implant an ICD, particularly in light of the VT recorded by the WCD.

While beyond the scope of the discussion in response to question no. 3, VT ablation directed toward the arrhythmia or premature ventricular contraction ablation as a trigger might have also been an appropriate option.

Once the decision to proceed with ICD implantation was made, it appears the clinicians favored placing a dual-chamber system over a single-chamber transvenous ICD or a subcutaneous ICD. The merits and rationale for a traditional transvenous ICD versus a subcutaneous ICD have been well-outlined by Al-Khatib et al.^33^ The forthcoming results of the Prospective, Randomized Comparison of Subcutaneous and Transvenous ICD Therapy (PRAETORIAN) trial (ClinicalTrials.gov identifier: NCT01296022) are expected to further inform our decision-making capacity in this regard.

Given the occurrence of VT prior to ICD implant, this patient is now considered a “secondary prevention” patient, not a “primary” one. One might favor implantation of a subcutaneous ICD at the time of implant, as the documented tachycardia of about 280 bpm (seen while wearing the WCD) is less likely to have responded to ATP. Importantly, that a subsequent slower VT occurred (successfully addressed by ATP) after implant of the dual-chamber device does not support the validity of the initial decision to implant a transvenous system. Irrespective of any post-hoc reasoning applied, the primary flaw of the current subcutaneous ICD remains the lack of ATP ability. Substantial evidence supports the view that most expected tachyarrhythmias can be readily addressed with ATP. Further, it is likely that the capability and strengths of ATP as a therapy have not been fully explored to date.^34,35^

As a personal preference, a device with ATP capability in the absence of compelling circumstances (e.g., patient preference, access issues) is favored over a subcutaneous device given the effectiveness of ATP. The advent of complex multicomponent systems to address the pacing and ATP deficiencies of the existing subcutaneous ICD is—in this clinician’s mind—a needlessly complex, expensive, and unwelcome mouse trap^36,37^ (see Figure 6 of Lau et al.^37^). In the future, a unified, single-system, nontransvenous ICD with ATP capability may provide a full range of tachyarrhythmia therapies (both ATP and shock) as well as backup rescue bradycardia pacing.^38^ In short, the choice of a full-featured traditional transvenous system for the patient described by Johnston et al.^1^ is recommended.

Parenthetically and despite the recent change in the Centers for Medicare & Medicaid Services’ payment policy,^39^ the MagnaSafe Registry,^40^ and Hopkins data,^41^ implantation of an MRI-conditional ICD should be strongly considered so that a patient might receive unfettered access to MRI in the future.^42^ Finally, given the patient’s age, selecting a pulse generator with the longest projected longevity,^43^ even if outside any contract restrictions of the implanting center, is likely to be in the patient’s best interest in order to minimize battery changes down the road.

In the absence of a clear pacing indication requiring atrioventricular synchrony and with optimal programming, little evidence to support the value of choosing a dual-chamber ICD system versus a single-chamber system exists,^44–46^ although some studies encouragingly favor dual-chamber configurations to reduce inappropriate shocks.^47^ Perhaps the team managing the patient was concerned about bradycardia if nadolol and sotalol were continued. From what is presented,^1^ the patient does not appear to need dual-chamber pacing or have any atrial arrhythmias of concern. Sweeney offers a useful approach to ICD selection and management.^48^ Either a single-chamber ICD or VDD^49^ defibrillator system are likely appropriate. As some have said informally, “as a rule of thumb, you should always put in an odd number of leads.”

Finally, paying attention to driving and exercise restrictions congruent with current guidelines and consensus documents is also important for this patient.

In the end, the case by Johnston et al. was an excellent choice to present and highlights the diagnostic and therapeutic challenges that clinicians might face. The opportunity to comment on the case was a privilege and quite welcome.

J. Rod Gimbel, md (gimbeljr@ix.netcom.com)^1^

^1^Ascension Medical Group, Mequon, WI, USA

The author reports no conflicts of interest for the published content.

References1.JohnstonSLAshwathMLondonBTorgersonJTosicAOlshanskyBThe mysterious case of an athletic woman with recurrent syncope and a “normal” heart.J Innov Cardiac Rhythm Manage.20191073744374910.19102/icrm.2019.100701PMC7252779324777412.BrignoleMMoyaAde LangeFJ2018 ESC Guidelines for the diagnosis and management of syncope.Eur Heart J.2018392118831948[CrossRef][PubMed]2956230410.1093/eurheartj/ehy0373.AlboniPBrognoleMMenozziCDiagnostic value of history in patients with syncope with or without heart disease.J Am Coll Cardiol.200137719211928[CrossRef][PubMed]1140113310.1016/s0735-1097(01)01241-44.OlshanskyBIs syncope the same thing as sudden death except that you wake up?J Cardiovasc Electrophysiol.199781010981101[PubMed]936381210.1111/j.1540-8167.1997.tb00995.x5.TesterDJAckermanMJThe molecular autopsy: should the evaluation continue after the funeral?.Pediatr Cardiol.2012333461470[CrossRef][PubMed]2230739910.1007/s00246-012-0160-8PMC33325376.AsatryanBSchallerASeilerJUsefulness of genetic testing in sudden cardiac arrest survivors with or without previous clinical evidence of heart disease.Am J Cardiol.20191231220312038[CrossRef][PubMed]3097543210.1016/j.amjcard.2019.02.0617.SaklaniPKrahnAKleinGSyncope.Circulation.20131271213301339[CrossRef][PubMed]2352953410.1161/CIRCULATIONAHA.112.1383968.RögerSRosenkaimerSLHohneckATherapy optimization in patients with heart failure: the role of the wearable cardioverter-defibrillator in a real-world setting.BMC Cardiovasc Disord.201818152[CrossRef][PubMed]2954444210.1186/s12872-018-0790-8PMC58560029.LiangJJBiancoNRMuserDEnriquezASantangeliPD’SouzaBAOutcomes after asystole events occurring during wearable defibrillator-cardioverter use.World J Cardiol.20181042125[CrossRef][PubMed]2970716410.4330/wjc.v10.i4.21PMC591988910.ZyllaMMHillmannHAKProctorTUse of the wearable cardioverter-defibrillator (WCD) and WCD-based remote rhythm monitoring in a real-life patient cohort.Heart Vessels.2018331113901402[CrossRef][PubMed]2972167410.1007/s00380-018-1181-x11.PrystowskyENPadanilamBJJoshiSFogelRIVentricular arrhythmias in the absence of structural heart disease.J Am Coll Cardiol.2012592017331744[CrossRef][PubMed]2257531010.1016/j.jacc.2012.01.03612.PedersenCTKayGNKalmanJEHRA/HRS/APHRS expert consensus on ventricular arrhythmias.Heart Rhythm.20141110e166e196[CrossRef][PubMed]2517948910.1016/j.hrthm.2014.07.02413.OwenHJBosMJAckermanMJWearable cardioverter defibrillators for patients with long QT syndrome.Int J Cardiol.2018268132136[CrossRef][PubMed]3004177710.1016/j.ijcard.2018.04.00214.PieterseAHStiggelboutAMMontoriVMShared decision making and the importance of time.JAMA.2019 Apr 19[Epub ahead of print]. [CrossRef][PubMed]10.1001/jama.2019.37853100233715.UbelPAAbernethyAPZafarSYFull disclosure—out-of-pocket costs as side effects.N Engl J Med.20133691614841486[CrossRef][PubMed]2413117510.1056/NEJMp130682616.OlginJEPletcherMJVittinghoffEWearable cardioverter-defibrillator after myocardial infarction.N Engl J Med.20183791312051215[CrossRef][PubMed]3028065410.1056/NEJMoa1800781PMC627637117.MoradyFBaermanJMDiCarloLAJrDeBuitleirMKrolRBWahrDWA prevalent misconception regarding wide-complex tachycardias.JAMA.19852541927902792[CrossRef][PubMed]405748818.DunckerDKonigTHohmannSBauersachsJVeltmannCAvoiding untimely implantable cardioverter/defibrillator implantation by intensified heart failure therapy optimization supported by the wearable cardioverter/defibrillator—the PROLONG study.J Am Heart Assoc.201761pii: e004512[CrossRef][PubMed]10.1161/JAHA.116.004512PMC55236342809609819.FranconeMChimentiCGaleaNCMR sensitivity varies with clinical presentation and extent of cell necrosis in biopsy-proven acute myocarditis.JACC Cardiovasc Imaging.201473254263[CrossRef][PubMed]2456021410.1016/j.jcmg.2013.10.01120.CaforioALPankuweitSArbustiniECurrent state of knowledge on aetiology, diagnosis, management, and therapy of myocarditis: a position statement of the European Society of Cardiology Working Group on Myocardial and Pericardial Diseases.Eur Heart J.20133433263626482648a2648d[CrossRef][PubMed]2382482810.1093/eurheartj/eht21021.GrunSSchummJGreulichSLong-term follow-up of biopsy-proven viral myocarditis: predictors of mortality and incomplete recovery.J Am Coll Cardiol.2012591816041615[CrossRef][PubMed]2236542510.1016/j.jacc.2012.01.00722.AquaroGDPerfettiMCamastraGCardiac MR with late gadolinium enhancement in acute myocarditis with preserved systolic function: ITAMY study.J Am Coll Cardiol.201770161977198710.1016/j.jacc.2017.08.0442902555423.PiersSREveraertsKvan der GeestRJMyocardial scar predicts monomorphic ventricular tachycardia but not polymorphic ventricular tachycardia or ventricular fibrillation in nonischemic dilated cardiomyopathy.Heart Rhythm.201512102106211410.1016/j.hrthm.2015.05.0262600494224.ZorziAPerazzolo MarraMRigatoINonischemic left ventricular scar as a substrate of life-threatening ventricular arrhythmias and sudden cardiac death in competitive athletes.Circ Arrhythm Electrophysiol.201697pii: e004229[CrossRef][PubMed]10.1161/CIRCEP.116.004229PMC49566792739021125.BeckerMAJCornelJHvan de VenPMvan RossumACAllaartCPGermansTThe prognostic value of late gadolinium-enhanced cardiac magnetic resonance imaging in nonischemic dilated cardiomyopathy: a review and meta-analysis.J Am Coll Cardiol Img.201811912741284[CrossRef][PubMed]10.1016/j.jcmg.2018.03.0062968035126.PerettoGSalaSRizzoSArrhythmias in myocarditis: state of the art.Heart Rhythm.2019165793801[CrossRef][PubMed]3047654410.1016/j.hrthm.2018.11.02427.HallidayBPBaksiAJGulatiAOutcome in dilated cardiomyopathy related to the extent, location, and pattern of late gadolinium enhancement.JACC Cardiovasc Imaging.2018pii: S1936-878X(18)30670-3[CrossRef][PubMed]10.1016/j.jcmg.2018.07.015PMC66826093021939728.GräniCEichhornCBièreLPrognostic value of cardiac magnetic resonance tissue characterization in risk stratifying patients with suspected myocarditis.J Am Coll Cardiol.2017701619641976[CrossRef][PubMed]2902555310.1016/j.jacc.2017.08.050PMC650684629.Schluz-MengerJDiagnostic accuracy of CMR in biopsy-proven acute myocarditis.JACC Cardiovasc Imaging.201473264266[CrossRef][PubMed]2465110010.1016/j.jcmg.2014.01.00530.Perazzollo MarraMThieneGRizzoSCardiac magnetic resonance features of biopsy-proven endomyocardial diseases.JACC Cardiovasc Imaging.201473309312[CrossRef][PubMed]2465110310.1016/j.jcmg.2013.10.01631.CooperLTBaughmanKLFeldmanAMThe role of endomyocardial biopsy in the management of cardiovascular disease. A Scientific Statement from the American Heart Association, the American College of Cardiology, and the European Society of Cardiology Endorsed by the Heart Failure Society of America and the Heart Failure Association of the European Society of Cardiology.Eur Heart J.2007282430763093[CrossRef][PubMed]1795962410.1093/eurheartj/ehm45632.CaforioALPChengCPerazzolo MarraMHow to improve therapy in myocarditis: role of cardiovascular magnetic resonance and of endomyocardial biopsy.Eur Heart J Suppl.201921Suppl BB19B22[CrossRef][PubMed]3094893710.1093/eurheartj/suz014PMC643989833.Al-KhatibSMFriedmanPEllenbogenKADefibrillators: selecting the right device for the right patient.Circulation.20161341813901404[CrossRef][PubMed]2779925710.1161/CIRCULATIONAHA.116.02188934.WatanabeTInoueKKashiwaseKEfficacy of anti-tachycardia pacing for terminating fast ventricular tachycardia in Japanese implantable cardioverter defibrillator patients. Primary results of the SATISFACTION study.Circ J.2014781126432650[CrossRef][PubMed]2526296310.1253/circj.cj-14-014635.AngueraIDallaglioPMartínez-FerrerJShock reduction with multiple bursts of antitachycardia pacing therapies to treat fast ventricular tachyarrhythmias in patients with implantable cardioverter defibrillators: a multicenter study.J Cardiovasc Electrophysiol.2015267774782[CrossRef][PubMed]2591681410.1111/jce.1269936.TjongFVYKoopBEThe modular cardiac rhythm management system: the EMPOWER leadless pacemaker and the EMBLEM subcutaneous ICD.Herzschrittmacherther Elektrophysiol.2018294355361[CrossRef][PubMed]3038234110.1007/s00399-018-0602-yPMC626740737.LauCPSiuCWTseHFFuture of implantable devices for cardiac rhythm management.Circulation.20141297811822[CrossRef][PubMed]2455055210.1161/CIRCULATIONAHA.112.00031238.BoersmaLVAMerkelyBNeuzilPTherapy from a novel substernal lead—the ASD2 study.JACC Clin Electrophysiol.201952186196[CrossRef][PubMed]3078468910.1016/j.jacep.2018.11.00339.Centers for Medicare & Medicaid Services. National Coverage Analysis (NCA) for Magnetic Resonance Imaging (MRI) (CAG-00399R4)Available at: https://www.cms.gov/medicare-coverage-database/details/nca-details.aspx?NCAId=289&bc=ACAAAAAAAAAAAA%3D%3D&. Accessed April 30, 201940.RussoRJCostaHSSilvaPDAssessing the risks associated with MRI in patients with a pacemaker or defibrillator.N Engl J Med.20173768755764[CrossRef][PubMed]2822568410.1056/NEJMoa160326541.MillerJDNazarianSHalperinHRImplantable electronic cardiac devices and compatibility with magnetic resonance imaging.J Am Coll Cardiol.2016681415901598[CrossRef][PubMed]2768720110.1016/j.jacc.2016.06.06842.GimbelJRPassmanRKanalEMRI-conditional devices, safety, and access: choose wisely and when you come to the fork in the road, take it.Pacing Clin Electrophysiol.2015381213731376[CrossRef][PubMed]2640357510.1111/pace.1275143.Von GuntenSSchaerBAYapSCLongevity of implantable cardioverter defibrillators: a comparison among manufacturers and over time.Europace.2016185710717[CrossRef][PubMed]2660907610.1093/europace/euv296PMC488011344.PokorneySDParzynskiCSDaubertJPTemporal trends in and factors associated with use of single- versus dual-coil implantable cardioverter-defibrillator leads: data from the NCDR ICD registry.JACC Clin Electrophysiol.201736612619[CrossRef][PubMed]2975943510.1016/j.jacep.2016.11.01445.FriedmanPABradleyDKoestlerCA prospective randomized trial of single- or dual-chamber implantable cardioverter-defibrillators to minimize inappropriate shock risk in primary sudden cardiac death prevention.Europace.2014161014601468[CrossRef][PubMed]2492894810.1093/europace/euu02246.PetersonPVarosyPDHeidenreichPAAssociation of single- vs dual-chamber ICDs with mortality, readmissions, and complications among patients receiving an ICD for primary prevention.JAMA.20133091920252034[CrossRef][PubMed]2367731410.1001/jama.2013.4982PMC375292447.KolbCStumerMSickPReduced risk for inappropriate implantable cardioverter-defibrillator shocks with dual-chamber therapy compared with single-chamber therapy: results of the randomized OPTION study.JACC Heart Fail.201426611619[CrossRef][PubMed]2528203310.1016/j.jchf.2014.05.01548.SweeneyMOThe implantable cardioverter-defibrillator minimalist: an approach to patient follow-up and management of implantable defibrillators.Circulation.20121263369377[CrossRef][PubMed]2280165410.1161/CIRCULATIONAHA.111.02388749.SafakESchmitzDKonorzaTWendeCDe Ros JoseOSchirdewanAClinical efficacy and safety of an implantable cardioverter-defibrillator lead with a floating atrial sensing dipole.Pacing Clin Electrophysiol.2013368952962[CrossRef][PubMed]2369226210.1111/pace.12171

## Dr. Knilans points out

The case presented by Johnston et al.^1^ illustrates several aspects to consider in the association between ventricular dysfunction and the risk of sudden death. These include the patient’s tolerance of tachyarrhythmia and the broadening appropriate use of WCDs in the management of and during the evaluation of ventricular arrhythmias and syncope.

Current guidelines for the management of ventricular arrhythmia appropriately focus on individuals with moderate to severe ventricular dysfunction, as this is the population who is most at risk in a society with a high frequency of coronary artery disease and myocardial ischemia and infarction. Those with normal ventricular function associated with cardiac ion channelopathies also receive their share of attention. There are few management recommendations published at this time for individuals with structurally normal hearts and normal ventricular function who have ventricular arrhythmias associated with other causes, including myocarditis. For the management of ventricular arrhythmia, individuals with myocarditis and a left ventricular ejection fraction (LVEF) of less than 35%, among other indications, meet the criteria for WCD (IIb recommendation). As the patient in the present report had a LVEF of more than 35%, she would not quality for a WCD under the current criteria.^2^ Of note, the recommendations do state that “patients with . . . myocarditis [among other listed conditions such as newly diagnosed nonischemic cardiomyopathy, recent myocardial infarction, and secondary cardiomyopathy] . . . are at increased risk of [VT]/sudden cardiac arrest. However, the WCD is of unproven benefit in these settings, in part because the clinical situation may improve with therapy and time.”^2^ It would seem to me that the WCD would be ideal in situations where the patient is expected to improve with therapy and time, as it can be applied in times of increased risk and easily withdrawn without complications.

Normal ventricular function (probably both systolic and diastolic function) in the described individual likely allowed her to survive multiple events of ventricular arrhythmia and indeed maintain consciousness with a ventricular rate approaching 300 bpm. Peripheral vasodilation plausibly resulted in her syncopal/presyncopal symptoms during exertion as compared with the event recorded by the WCD. There is a widespread belief that VT inherently results in more hemodynamic compromise than supraventricular arrhythmia (SVT). Over the years, I have seen many ECG tracings of wide QRS complex tachycardias in young individuals (many with VT) with the words “SVT, blood pressure stable” handwritten at the top, confirming this notion. VT may present with a greater likelihood to cause compromise due to a higher probability of atrioventricular dissociation than SVT. However, there is no other a priori reason for VT to lead to more instability than SVT conducted with aberration or preexcitation at the same rate in individuals with normal ventricular function. This myth is propagated by the preponderance of adult patients with VT having depressed ventricular function as compared with the population with SVT, usually having normal function. One could argue that normal diastolic function is as or more important than systolic function in maintaining hemodynamic stability at extremely fast heart rates. The reported patient’s prior history of high-level athletic performance (as an ex–college basketball player) and likely continued high activity levels (as suggested by her kayaking with her daughter) as well as good cardiometabolic function probably contributed to her tolerance of the arrhythmia.

At the time of placement of the WCD in the reported individual, the diagnosis of myocarditis was suspected but not confirmed and she was awaiting a cardiac MRI scan. Given her syncopal event and documented ventricular arrhythmia, she would seem to meet United States Food and Drug Administration approval guidelines for WCD usage, in that the device can be used “for patients 18 years of age and older who are at risk for sudden cardiac arrest and [who] are not candidates for or [who] refuse an implanted defibrillator.”^3^ Current published guidelines allow for the use of a WCD under the recommendation that the “use of WCDs may be reasonable when there is concern about a heightened risk of SCD that may resolve over time.”^4^ The use of a WCD in an individual with myocarditis and normal ventricular function is hardly new and was reported a decade ago as a successful strategy in a similar case, albeit in the presence of acute myocarditis.^5^ Use of a WCD in these situations seems prudent and is likely cost-effective.

As the reported patient survived prior episodes and was conscious during the arrhythmia detected with the WCD, it is possible that a simple cardiac event monitor could have established the same diagnosis and achieved the same end-result. However, there was a very real possibility for the arrhythmia to degenerate and result in a fatal outcome. Once the diagnosis was confirmed by MRI imaging, I believe that the placement of an ICD was appropriate. The documented ventricular arrhythmia had the potential to be life-threatening and, because the myocarditis was in the chronic stage, the arrhythmia was unlikely to resolve within a predictable time-course, if ever. Use of antiarrhythmic drug therapy alone seems to carry an inappropriately high risk in the context of this case. Catheter ablation of the tachycardia could also be considered, but longer-term reliability in this setting would be uncertain. Thought could have also been given to the placement of a subcutaneous device, but the successful use of antitachycardia pacing in this patient clearly illustrates a benefit of a transvenous approach. The value of an atrial lead is more difficult to argue and a single-chamber device may have suited the patient’s needs with a somewhat lower degree of risk and cost.

Timothy K. Knilans, md (timothy.knilans@cchmc.org)^1,2^

^1^Department of Pediatrics, Division of Cardiology, Cincinnati Children’s Hospital Medical Center, Cincinnati, OH, USA

^2^University of Cincinnati College of Medicine, Cincinnati, OH, USA

The author reports no conflicts of interest for the published content.

References1.JohnstonSLAshwathMLondonBTorgersonJTosicAOlshanskyBThe mysterious case of an athletic woman with recurrent syncope and a “normal” heart.J Innov Cardiac Rhythm Manage.20191073744374910.19102/icrm.2019.100701PMC7252779324777412.Al-KhatibSMStevensonWGAckermanMJ2017 AHA/ACC/HRS guideline for management of patients with ventricular arrhythmias and the prevention of sudden cardiac death: a report of the American College of Cardiology Foundation/American Heart Association Task Force on Clinical Practice Guidelines and the Heart Rhythm Society.J Am Coll Cardiol.20187214e9122010.1016/j.jacc.2017.10.054290972963.United States Food and Drug AdministrationPremarket Approval for LifeVest Wearable DefibrillatorAvailable at: https://www.accessdata.fda.gov/scripts/cdrh/cfdocs/cfpma/pma.cfm?id=P010030S056. Accessed April 30, 20194.PicciniJPAllenLAKudenchukPJWearable cardioverter-defibrillator therapy for the prevention of sudden cardiac death: a science advisory from the American Heart Association.Circulation.20161331717151727[CrossRef][PubMed]2702206310.1161/CIR.00000000000003945.ProchnauDSurberRKuehnertHSuccessful use of a wearable cardioverter-defibrillator in myocarditis with normal ejection fraction.Clin Res Cardiol.2010992129131[CrossRef][PubMed]1992129710.1007/s00392-009-0093-2

## Dr. Mirro and Mr. Zirille emphasize

This case underscores the prime importance of obtaining a detailed history in patients presenting with syncope. The American College of Cardiology/American Heart Association/Heart Rhythm Society syncope treatment guidelines reinforce this recommendation, classifying syncope as class 1B-NR (level of evidence: moderate quality; data from nonrandomized trials).^1^ The suspicion of a near-fatal syncopal episode during exertion in a relatively healthy individual with corrected QT prolongation (hypokalemia) further underscores the importance of either hospitalization or use of the WCD. Previous research has documented that hospitalization rarely results in a determination of the etiology of syncope,^2^ while the elucidation of an etiology at discharge is even less likely when patients are admitted for one-day inpatient stays or to emergency department observation units.^3^ Therefore, we agree completely with the decision made by Johnston et al.^4^ to pursue emergency department discharge and use of the WCD. As inherited channelopathy was the suspected diagnosis prior to cardiac MRI, the use of a provocative electrophysiologic study would not have been appropriate. The aforementioned syncope guidelines^1^ support the use of monitoring technology, and the pathway calls for a wearable monitor or use of an implantable loop recorder. The WCD boasts an electrocardiogram-monitoring system as a looping recorder, which can be routinely monitored remotely by clinics.^5^ However, many clinicians are unaware of the monitoring capability of the device and consider it merely a wearable automatic external defibrillator. In this complex case, we applaud these clinicians for placing the patient at the center of their decision-making process and considering the nuances of her history in their treatment plan. The use of medical practice guidelines has always been promoted as a pathway but not a dictum, and should be applied based on the individualization of clinical circumstances.

In further considering this case, it can be said that the preservation of cerebral blood flow with a 300-bpm rate in a healthy individual is an excellent example of cerebral autoregulation that can preserve consciousness for a short period of time.^6^ Also, the detection of monomorphic VT by the WCD, although at a rate of 300 bpm, suggested that a transvenous ICD with the ability to perform ATP would have been the best choice for this patient. As demonstrated by this case, the use of antiarrhythmic drug therapy later on slowed the spontaneous VT rate, allowing ATP therapy to be successful and confirming the validity of the clinical approach to her care that was adopted. Finally, the identification of a discrete scar and monomorphic VT suggests that VT ablation could perhaps be considered for this patient in the future, particularly if recurrent VT is an ongoing issue.

Michael J. Mirro, md, fhrs, facc, faha (michael.mirro@parkview.com)^1,2^ and Francis M. Zirille, ba^1,2^

^1^Parkview Mirro Center for Research and Innovation, Parkview Health, Fort Wayne, IN, USA

^2^Indiana University School of Medicine, Indianapolis, IN, USA

Dr. Mirro reports he is a trustee for Indiana University; has equity interest (public) in iRhythm; is a consultant for Zoll Medical Corporation; and has received research grants from Medtronic, Biotronik, and Janssen Pharmaceutica. Mr. Zirille reports no conflicts of interest for the published content.

References1.ShenWKSheldonRSBendittDG2017 ACC/AHA/HRS guideline for the evaluation and management of patients with syncope: a report of the American College of Cardiology/American Heart Association Task Force on Clinical Practice Guidelines and the Heart Rhythm Society.J Am Coll Cardiol.20177016e3911010.1016/j.jacc.2017.03.003282862212.ShinTGKimJSSongHGStandardized approaches to syncope evaluation for reducing hospital admissions and costs in overcrowded emergency departments.Yonsei Med J.201354511101118[CrossRef][PubMed]2391855910.3349/ymj.2013.54.5.1110PMC37431983.GrossmanAMVolzKAShapiroNIComparison of 1-day emergency department observation and inpatient ward for 1-day admissions in syncope patients.J Emerg Med.2016502217222[CrossRef][PubMed]2668284710.1016/j.jemermed.2015.06.0134.JohnstonSLAshwathMLondonBTorgersonJTosicAOlshanskyBThe mysterious case of an athletic woman with recurrent syncope and a “normal” heart.J Innov Cardiac Rhythm Manage.20191073744374910.19102/icrm.2019.100701PMC7252779324777415.KleinHUGoldenbergIMossAJRisk stratification for implantable cardioverter defibrillator therapy: the role of the wearable cardioverter-defibrillator.Eur Heart J.2013342922302242[CrossRef][PubMed]2372969110.1093/eurheartj/eht1676.AaslidRLindegaardKFSortebergWNornesHCerebral autoregulation dynamics in humans.Stroke.19892014552[CrossRef][PubMed]249212610.1161/01.str.20.1.45
